# Diabetes related foot disease; ‘know thine enemy’

**DOI:** 10.1186/1757-1146-4-S1-O8

**Published:** 2011-05-20

**Authors:** Shan M Bergin, Caroline A Brand, Peter G Colman, Donald A Campbell

**Affiliations:** 1 Podiatry Department, Dandenong Hospital, Melbourne, Victoria, 3172, Australia; 2 Clinical Epidemiology and Health Service Evaluation Unit, Royal Melbourne Hospital, Melbourne, Victoria, 3052, Australia; 3 Department of Diabetes and Endocrinology, Royal Melbourne Hospital, Melbourne, Victoria, 3052, Australia; 4 Department of General Medicine, Monash University, Melbourne, Victoria, 3169, Australia

## Background

Information describing variation in health outcomes for individuals with diabetes related foot disease (DRFD), across socioeconomic strata is lacking. Focussing on the clinical aspects of foot disease, in individuals with DRFD that reside in areas of known social disadvantage, may not result in the desired clinical outcomes. The aim of this study was to investigate variation in rates of hospital separations for DRFD and the relationship with levels of social advantage and disadvantage.

## Methods

Using the Index of Relative Socioeconomic Disadvantage (IRSD) each Local Government Area (LGA) across Victoria was ranked from most to least disadvantaged. Those LGAs ranked at the lowest end of the socioeconomic scale (Group A) were compared with those at the higher end of the scale (Group B) in terms of total and per capita hospital separations for peripheral neuropathy, peripheral vascular disease, foot ulceration, cellulitis, osteomyelitis and amputation. Hospital separations data was compiled from the Victorian Admitted Episodes Database (VAED).

## Results

Total and per capita separations were 2,268 (75.3/1,000) and 2,734 (62.3/1,000) for Groups A and B respectively. Most notable variation was for foot ulceration (Group A 18.1/1,000 Vs Group B 12.7/1,000, rate ratio 1.4, 95%CI 1.3, 1.6) and below knee amputation (Group A 7.4/1,000 Vs Group B 4.1/1,000, rate ratio 1.8 95%CI 1.5, 2.2). Males recorded a greater overall number of hospital separations across both socioeconomic groups with 66.2% of all separations for Group A and 81.0% of all separations for Group B recorded by males. However, when comparing mean age, males form Group A tended to be younger when compared with males from Group B (mean age 53 years Vs 68.7 years).

## Conclusions

Variation appears to exist for hospital separations for DRFD across socioeconomic strata. Specific strategies should be incorporated into health policy and planning to combat disparities between social status and health outcomes.

